# Intensive care admission after cardiac arrest: cardiac versus noncardiac causes and consequences for treatment

**DOI:** 10.1186/cc12245

**Published:** 2013-03-19

**Authors:** T Hargreaves, H Kingston, M Crews, M Mogk, I Welters

**Affiliations:** 1 Royal Liverpool University Hospital, Liverpool, UK; 2 Moredata GmbH, Giessen, Germany; 3 Liverpool University, Liverpool, UK

## Introduction

In-hospital (IHCA) and out-of-hospital cardiac arrest (OHCA) are associated with high mortality [1]. Studies suggest that up to 68% of OHCA is due to acute coronary syndrome, with 38% requiring percutaneous coronary intervention (PCI) [1]. However, revascularisation may not always be available or address the underlying pathology. This study aimed to establish the prevalence of different aetiologies for IHCA and OHCA, and the use of emergency treatment for these patients.

## Methods

A retrospective case-note review of all patients admitted between 2008 and 2011 to the ICU of an inner-city university hospital after OHCA or IHCA. Biometric data, presenting cardiac rhythm, presumed cause of arrest, management and outcomes were recorded. The Kruskal-Wallis test was used for numerical data analysis and chi-square test for categorical data.

## Results

Data were analysed for 64 patients - 44 (69%) following OHCA and 20 (31%) after IHCA. The median APACHE score for OHCA was 17 and for IHCA was 23.5 (*P *= 0.001). Hospital survival rate was 10% (*n = *2) for IHCA and 38.6% (*n = *17) for OHCA (*P <*0.02). A total of 34.1% (*n = *15) OHCA were due to myocardial infarction (MI) compared with 10% (*n = *2) of IHCA (*P <*0.05). The most prevalent aetiologies were MI (*n = *17), hypoxia (*n = *10), cardiac other (*n = *5), sepsis (*n = *4), arrhythmia (*n = *3) and PE (*n = *3). In two IHCA patients more than one likely cause of arrest was reported and in 19 cases no cause was identified. The presenting rhythm was ventricular fibrillation (VF) in 45.3% (*n = *29), pulseless electrical activity in 32.8% (*n = *21) and asystole in 20.3% (*n = *13). A total of 9.4% (*n = *6) were thrombolysed and one (1.6%) patient was referred for emergency PCI.

## Conclusion

As previously reported [2], IHCA was associated with a worse prognosis than OHCA. The OHCA survival rate was better than reported elsewhere [3]. The percentage of IHCA attributed to MI was low. Only one OHCA patient was referred for emergency PCI. Routine coronary angiography with *ad hoc *PCI in VF OHCA has been associated with increased survival [4]. Greater availability of PCI post OHCA could further improve mortality in patients with a primary cardiac pathology. Further investigation should include management of noncardiogenic cardiac arrest.
